# Global Patterns of Agricultural Investment and Food Security: Evidence from the fDi Markets Database

**DOI:** 10.3390/foods12091827

**Published:** 2023-04-28

**Authors:** Yongzhi Zhao, Yangfen Chen

**Affiliations:** 1 Institute of Agricultural Economics and Development, Chinese Academy of Agricultural Sciences, Beijing 100081, China; yongzhi.zhao12@outlook.com; 2 Environmental Economics and Natural Resources Group, Department of Social Sciences, Wageningen University and Research, 6706 KN Wageningen, The Netherlands

**Keywords:** agricultural investment, food security, investment motivation, COVID-19, per capita protein intake

## Abstract

The number of hungry people is on the rise and more efforts are needed to improve the global food security status. The Food and Agriculture Organization (FAO) proposes more investment in the agricultural sector to boost production and alleviate hunger. However, there are fewer papers that distinguish enterprises investment from public investment. In this case, we take advantage of detailed investment data in the fDi Markets database to explore the global patterns of agricultural investment. In particular, we identify the top destination countries based on aggregate and sub-sectoral agricultural investment data. Then we investigate the relationship between agricultural investment and food security, which is measured by per capita protein intake. Finally, we propose some suggestions from the investment motivation perspective to help food-insecure countries to attract overseas investment. We find that developed countries are the primary sources of global agricultural investment and these sources have been becoming more diverse in the past decade. It implies the trend towards a more inclusive investment environment worldwide. However, the global distribution of agricultural investment is uneven as food-insecure countries only receive 20% of the global agricultural investment. The top three destination countries, USA, China, and Russia, have a relatively high food security level. In contrast, countries suffering from food insecurity receive fewer investment projects, and most of which are on a small scale. Given the limited socio–economic development status in food-insecure countries, it is essential for all levels of society to help them and contribute to ending hunger.

## 1. Introduction

Food security is a crucial aspect of human well-being. There are only seven years remaining to achieve the Sustainable Development Goal (SDG) 2 “Zero Hunger”, but the task is becoming more challenging due to factors such as climate change, regional conflicts, and economic shocks. According to *The Sustainable Development Goals Report 2022*, an increasing number of people have been affected by hunger since 2014 and the COVID-19 pandemic makes it worse [1]. The FAO [2] estimated that more than 700 million people suffered from hunger in 2021, an increase of 46 million since 2020 and 150 million more than that in 2019. In particular, people affected by severe food insecurity increased in 2021, accounting for 11.7% of the global population, which highlights the impacts of COVID-19 on the most vulnerable people. Regarding the nutrition status worldwide, rural residents are suffering from various forms of malnutrition while urban residents are exposed to a higher risk of overweight and obesity. In 2020, 22% (149.2 million) of children under the age of five were affected by stunting and 6.7% (45.4 million) suffered from wasting. The prevalence of overweight among children under five increased to 5.7% (38.9 million) in 2020 [2]. Additionally, high food prices have been affecting many countries since 2016. The crisis in Ukraine adds further uncertainty to the affordability of a healthy diet since both Ukraine and Russia are major agricultural producers and exporters [3]. For example, the crisis affects the planting, harvesting, and transportation in Ukraine, which leads to a reduction in the supply market [4]. The global supply chains have been disrupted by the conflicts and agricultural trade has been hampered, especially the export from Russia and Ukraine [5]. Moreover, agricultural input costs have experienced a significant rise due to the conflicts between Ukraine and Russia, which negatively impact crop yields [4,6]. Given the growing threats to global food security, it is essential that all levels of society pay closer attention and that interdisciplinary solutions are sought urgently.

Various actions have been implemented to improve food security. Governments propose various policies to improve food security, such as food price controls and agricultural subsidies for small-scale farmers, and preferential tariffs for agricultural enterprises [7]. Non-government organizations keep investing to improve food and nutrition for the most vulnerable. Some cross-sectors collaborate to improve food security, such as public–private partnerships [8]. The FAO [9] emphasizes the engagement of small- and medium-sized enterprises in the agrifood systems transformation. Many papers also argue the positive effects of investment on food security. However, enterprises’ investment is usually motivated by maximizing profit, which is not necessarily consistent with the goal of food security improvement. How to guide agricultural investment towards food insecure countries and take advantage of these investments to improve food security is worthy of attention.

Responding to the FAO’s call and contributing to SDG Target 2, this paper explores the relationship between agricultural investment and food security. Specifically, we first focus on the global patterns of agricultural investment and identify the most popular destinations for agricultural investment projects. Then, based on the food security indicators, we explore the relationship between agricultural investment and food security. Finally, we propose some suggestions from the investment motivation perspective to help food-insecure countries attract more investment. We also call for efforts from all levels of society to improve food and nutrition status worldwide.

## 2. Literature Review

Food security is a hot topic in the literature and involves various aspects of socio–economic development. Both developing countries and developed country face food security issues but manifest in different ways. In developing countries, hungry people usually cannot afford sufficient, safe, and healthy food, while food insecurity usually refers to overweight and obesity in developed countries [2,10]. To improve the food security level, some papers focus on general agricultural interventions and others investigate the impacts of certain improvement projects. Bizikova, et al. [11] find input subsidies, cash transfers, food vouchers, and extension services are effective interventions to improve food security at an individual level. However, the performance of these interventions also varies across different design and operating plans. Rana et al. [12] highlight crop management strategies, such as utilizing fertilizers, caring for the seasonality, and introducing control systems to increase yield productivity based on the literature review from the botanical perspective. Deligios et al. [13] introduce an irrigation water management system that combines evaporative cooling practice with precision irrigation technique, which increases yields and improves water productivity. Shamah-Levy et al. [14] focus on food security governance at national level and point out that fragmentation of governance agencies and oligopoly problems in the agricultural sector threaten food security in Mexico. West et al. [15] evaluate one specific intervention program, OzHarvest’s six-week Nutrition Education and Skills Training program, and suggest that ensuring all citizens’ access to nutritious food is the key to improve food security. Furthermore, another strand of literature investigates the determinants of food security aiming at explaining the underlying mechanism. Feleke et al. [16] focus on household food security and find that technology adoption, farm size, and land quality could positively affect household food security. Allee et al. [17] explore national determinants and find that per-capita cereal production, governance level, and logistics performance are key drivers of food security improvement.

Food security measurement is the key to conducting research related to food security. Because food security is a relatively broad concept and includes four dimensions: access, availability, utilization, and stability according to the FAO’s definition. Scholars usually choose different indicators to capture different dimensions of food security based on their research objectives. Some papers develop their own indicators to measure more site-specific food security [18,19]. Based on the existing indicators, there are mainly two types of evaluation methods: micro-level and macro-level methods. At the macro level, that is at the national level, the most common indicators include the Global Hunger Index (GHI), the Food Insecurity Experience Scale (FIES), the prevalence of undernourishment, and food demand and supply [17,20,21]. The other kind is a household or individual indicator, such as nutrients intake (calorie, carbohydrates, and protein), dietary diversity, and food distribution within the family [22,23,24,25,26]. These micro-level indicators are usually used to measure food utilization and food stability. In this paper, our focus is the relationship between agricultural investment and food security in the country dimension. To make the indicators comparable across developing and developed countries, we take advantage of national indicators. Specifically, we choose the per capita protein intake indicator as the measurement of food security following Katz-Rosene et al. [27] and Andriamparany et al. [28].

Regarding the discussion on investment and food security, many papers have provided evidence of the positive effects of agricultural investment on food security improvement. The underlying mechanism is mainly explained through three channels. First, investment improves food production efficiency and positively affects food security in host countries. The investment brings capital directly and improves agricultural Total Factor Productivity (TFP) through the spillover effect of knowledge and technology [29,30]. For example, large scale land investments bring modern agricultural techniques and close the crops yield gaps in host country, which contributes to the increase in food production and feeds more people in the host country [31]. Furthermore, increased agricultural production directly improves household consumption, including the intake of protein, vitamins, and carbohydrates [32]. Second, investment would promote economic development at the national level, and therefore provide employment opportunities for local residents [33,34]. Large-scale land investments could provide agricultural and non-agricultural jobs when it matches with local community [32,35]. Investment in commercial farms helps small-holders get access to modern inputs and improve their income levels [36]. Third, some studies suggest that agricultural investment positively affects food supply chains in host countries, which would promote regional and international trade in agricultural sectors [37].

However, the existing papers on investment and food security mainly focus on land investment and public investment. This is because the food security is usually considered as a public well-being issue and requires more effort from the public sector. As the important part of the global economy, the private sector (the enterprises) should also contribute to the improvement of global food security, especially during the recovering of the global economy from the COVID-19 pandemic. However, fewer papers distinguish enterprises’ investment from public investment and investigate its impacts on food security. To fill this gap and further contribute to the literature on food security, we focus on global agricultural investment that was invested by international enterprises and explore its relationship with food security indicators measured by per capita protein intake.

## 3. Data and Methods

Data on investment are usually limited due to commercial companies considering investment information highly confidential [38]. The most widely used databases in this field are the Land Matrix and the fDi Markets. The Land Matrix database has provided investment information on large-scale land deals in low- and middle-income countries since 2000. The fDi Markets database covers cross-border greenfield investments worldwide, including details on investment amount, project time, source country, and destination country. Each of them has its own advantages. For example, Land Matrix aims at monitoring large-scale land transactions, also known as “large-scale land acquisitions”. They categorize land deals, according to the objectives, into food crop, tourism, timber plantation, and the processing industry. However, the targeted areas in the Land Matrix database are low- and middle- income countries. By contrast, the geographical and sectoral focuses of the fDi Markets are larger. This database tracks investment projects across all sectors and almost all countries globally. The source is identified by the location of the global headquarters and the destination is geographical information on the project. The sectoral classification in fDi Markets is aligned with the North American Industry Classification System (NAICS) 2007 and could provide us sub-sectoral investment information to investigate detailed investment patterns within the agricultural sector. Specifically, we screen investment in the food and tobacco sector from January 2003 to June 2019 given the data availability. We obtain a total of 7135 investment transactions involving 19 sub-sectors.

Based on these agricultural investment transaction data obtained from the fDi Markets database, we identify the top source and destination countries of agricultural investment, and we approach this from three perspectives. Firstly, we analyze the top countries based on the aggregate value of agricultural investment from 2003 to 2019. Secondly, we examine the changes in top source and destination countries over time using annual agricultural investment data. We present data for four time points: 2003, 2008, 2013, and 2018. Thirdly, we categorize agricultural investment into two sub-sectors: agricultural production and agricultural processing, and identify the top source and destination countries within each sub-sector from 2003 to 2019. In Section 4.3, we provide a detailed classification of agricultural investment sub-sectors. By presenting these findings, we aim to provide insights into the trends and patterns of global agricultural investment. Once we have identified the top source and destination countries for agricultural investment, we proceed to compare the food security levels in these countries and explore the underlying correlation between investment and food security. To ensure clarity in the presentation of our data, we exclude transactions worth less than $60 million from our analysis and present the resulting investment flows using alluvial diagrams in Section 4.2. It is important to note that this exclusion does not affect our results, as our focus is on the top destination countries.

We obtain per capita protein intake (g/cap/day) data from FAOSTAT and further explore its relationship with investment. To match the aggregate agricultural investment, we take the average value of per capita protein intake from 2003 to 2019 for each country. We use a bubble chart to explore the relationship between agricultural investment and food security. In the bubble chart, different bubbles represent different countries and the larger bubble indicates a higher value of aggregate agricultural investment (USD million). The countries with higher protein intake are located in the further right and the countries receiving a bigger number of agricultural investment projects are located in the upper position. We can tell the basic relationship between agricultural investment and food security by comparing the location and size of different bubbles. The bubble chart allows us to focus on the amount of agricultural investment projects and the aggregate value of all investment projects at the same time, which provides us with more information on the relationship between investment and food security.

## 4. Results

There is around $630 billion in investment support in the food and agricultural sector annually from 2013 to 2018 [2]. The investment projects from the private sector, according to the fDi Markets database, accounted for $277 billion in total from 2003 to 2019, which involved 7134 transactions associated with food and tobacco and distributed in 159 countries and regions.

### 4.1. The Top Source and Destination Countries

As shown in Table 1, the main sources of global agricultural investment come from developed countries. The largest source country is USA, accounting for 19.1% ($52.9 billion) of the world’s agricultural investment. Switzerland and Germany also contribute to 8.6% ($23.9 billion) and 8.5% ($23.4 billion) of global agricultural investments respectively. Other notable sources include the UK (6.4%, $17.8 billion), Japan (5.2%, $14.4 billion), China (4.8%, $13.4 billion), and France (4.3%, $11.8 billion). These seven countries account for over half of global investment (56.8%, about $157.5 billion).

Regarding the destinations of agricultural investment, we can see a relatively dispersed pattern and main recipients are traditional agricultural countries. The total number of destinations is also more than the sources. Russia leads with 9.7% of total global agricultural investment, amounting to $26.8 billion. The second largest destination country is China, receiving $23.2 billion (8.4%). The following countries are USA (8.0%, $22.1 billion), the United Kingdom (4.4%, $12.2 billion), India (3.3%, $9.2 billion), Brazil (3.2%, $8.9 billion), Indonesia (2.9%, $8.0 billion), and Mexico (2.9%, $7.9 billion). The aggregate investment flowing into these eight countries is $118.3 billion, accounting for 42.7% of global agricultural investment.

### 4.2. Top Countries Varying with Years

**Figure 1:**
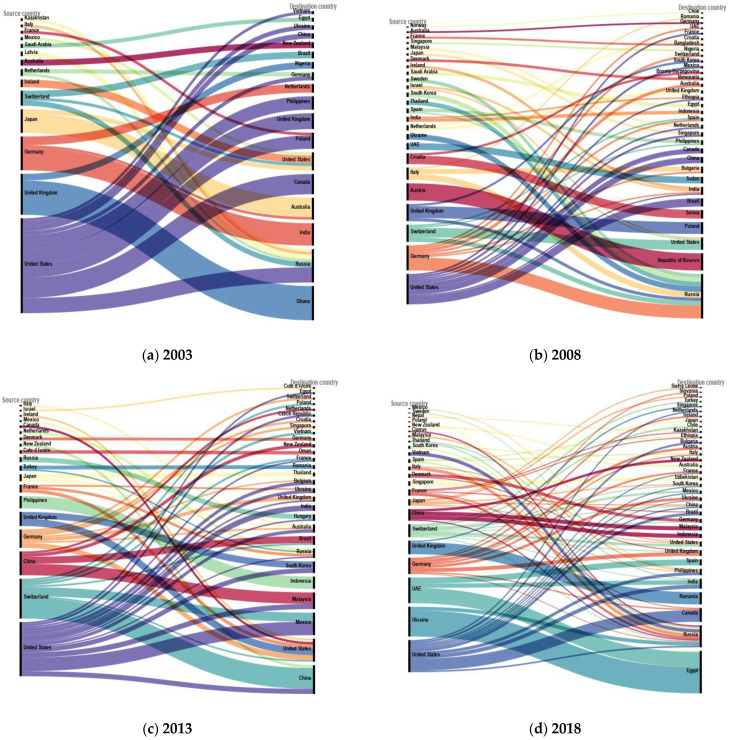
Agricultural investment flows in different years. Data is collected from the fDi Markets database.

The results in Figure 1 show that USA was the largest source country in 2003, 2008, 2013 and 2018. The gap between the top two largest source countries has been narrowing down. In 2003, USA was the largest source country with $2121.0 million in investment, and it was almost three times of the agricultural investment from the United Kingdom ($914.8 million), as the second largest source country. Germany and Japan were also important source countries with investments of $755.1 million and $529.3 million, respectively.

Regarding the top destination countries in 2003, Ghana was the largest destination country and received $764.8 million in investment that all came from the United Kingdom. Russia, as the second largest destination country, received $745.8 million totally and about 46% of the investment came from USA. India, Australia, and Canada were also top destination countries in 2003 and their investment mainly came from one single country. In 2008, the largest destination country, Russia, had more diverse sources of agricultural investment. The Republic of Kosovo appeared in the second place on the list of destination countries in 2008 due to a huge transaction worth $776 million from Austria.

The rankings of top source countries and destination countries have changed significantly between 2003 and 2018. In 2003, USA was the top source country, followed by the U.K. and Germany, while Ghana was the largest destination country, followed by Russia. By 2013, China had risen to the third position on the list of source countries and became the largest destination country. Switzerland rose to second place on the list of source countries in 2013 from number five in 2003. There are many new names at the top of the destination list in 2013, such as Mexico, Indonesia, Malaysia, and South Korea; we rarely saw them before. In 2018, more new countries appeared on the list of source and destination countries. The U.S. remained the largest source country, with Ukraine and the United Arab Emirates (UAE) following closely behind. Other important sources in 2018 included Germany, the U.K., Switzerland, and China. Egypt became the largest destination country in 2018 due to the investment from Ukraine and the United Arab Emirates (UAE). During this period, we can see a relative increase in diversity of investment sources and destinations. Investors become more inclined to choose multiple destinations to reduce market risks. On the other hand, destination countries become more open to accept foreign agricultural investment projects, which is consistent with the proposal by the UN on a “more inclusive investment environment”.

### 4.3. Top Countries Varying with Sub-Sectors

According to the classification of the Food and Tobacco Industry by the fDi Markets database, we categorize 19 sub-sectors into two groups: agricultural processing and agricultural production. Specifically, agricultural processing sector involves eight sub-sectors, namely animal slaughter and processing, bakeries and tortillas, food and beverage stores (food and tobacco), food services, seasonings and dressing, snack food, sugar and confectionary products, and wholesale trade (food and tobacco). The remaining 11 sub-sectors are classified as agricultural production, including tobacco, seafood products, grains and oilseed, fruits and vegetables and specialist foods, fishing hunting and trapping, dairy products, crop production, coffee and tea, animal production, animal food, and all other food.

The investment associated with agricultural processing amounts to $102.2 billion, involving a total of 2757 transactions and flowing from 87 source countries to 130 destination countries. Table 2 show that these investment projects mainly came from USA ($19.7 billion, 19.2%), Germany ($18.0 billion, 17.7%), Switzerland ($7.1 billion, 7.0%), the United Kingdom ($6.6 billion, 6.4%), and Japan ($5.5 billion, 5.4%). These five countries account for 56% of global agricultural processing investment, which indicates a concentrated investment pattern.

The U.S. is the largest source country of processing investment with $19.7 billion from 2003 to 2019. The most popular sub-sectors are sugar and confectionary products ($8.2 billion), food and beverage stores (food and tobacco) ($4.4 billion), snack food ($3.0 billion), and animal slaughtering and processing ($1.9 billion). Germany, as the second-largest source country, mainly invests in food and beverage stores ($13.4 billion, 74.5%), animal slaughtering and processing ($1.6 billion, 8.9%), and sugar and confectionary products ($1.4 billion, 7.7%).

As for the destination countries, the investment pattern is more scattered compared to the source countries. The top seven destination countries are USA ($9.0 billion, 8.8%), Russia ($8.2 billion, 8.0%), China ($7.6 billion, 7.4%), the United Kingdom ($6.6 billion, 6.4%), India ($4.8 billion, 4.7%) Germany ($3.7 billion, 3.7%), and Poland ($3.7 billion, 3.6%).

There are $175.0 billion invested in the agricultural production sector, involving a total of 4378 transactions, flowing from 93 source countries to 149 destination countries. As shown in Table 3, investment mainly came from USA ($33.2 billion, 19.0%), Switzerland ($16.8 billion, 9.6%), China ($11.4 billion, 6.5%), the United Kingdom ($11.2 billion, 6.4%), Japan ($8.8 billion, 5.1%), France ($8.7 billion, 5.0%), Thailand ($6.8 billion, 3.9%), and Vietnam ($6.4 billion, 3.7%). The top eight source countries accounted for 59% of investment in this sector.

As for the destination countries, the top five were Russia ($18.7 billion, 10.7%), China ($15.6 billion, 9.0%), USA ($13.1 billion, 7.5%), the Philippines ($6.6 billion, 3.8%), and Indonesia ($6.1 billion, 3.5%). Russia received most investment in the agricultural production sector, including $8.4 billion (45.1%) in the dairy products sub-sector and $2.3 billion (12.3%) in animal production. Other sub-sectors with more than $1 billion in investment are animal food ($1.7 billion, 9.3%), grain and oilseed ($1.6 billion, 8.3%), coffee and tea ($1.4 billion, 7.5%), and fruits, vegetables and specialist foods ($1.1 billion, 6.0%). China, as the second largest destination country, received most investment in animal food ($2.6 billion,16.4%) in the production sub-sector. Other main sub-sectors related to agricultural production include animal production ($2.0 billion, 12.7%), coffee and tea ($1.9 billion, 12.3%), crop production ($1.8 billion, 11.5%), and dairy products ($1.5 billion, 9.5%).

### 4.4. Food Security in the Main Destination Countries

**Figure 2:**
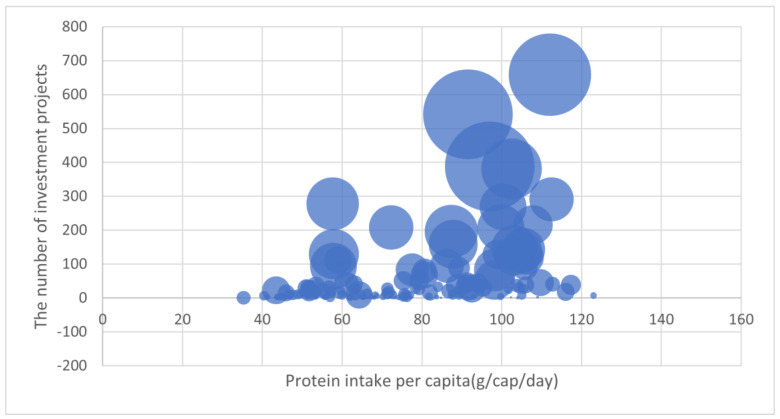
Food security and agricultural investment. Data comes from FAOSTAT.

As shown in Figure 2, we can see that the larger bubbles are mainly concentrated in the upper right side of the figure and the smaller bubbles are usually close to the horizontal line. This means that larger agricultural investment projects are usually located in countries with higher protein intake while small-size investments flow into countries with low protein intake. Second, this figure above can be roughly divided into two parts using a protein intake of 70 g/cap/day. If we define food-secure countries as countries with protein intake over 70 g/cap/day and otherwise as food-insecure countries, we can see a rough “80–20 rule”. Around 81% of agricultural investment flowed into food-secure countries while only 19% flowed into food-insecure countries. It suggests the quite uneven distribution of global agricultural investment. For example, USA, China, and Russia are the top three destination countries, whether considering the aggregate agricultural investment or sectoral agricultural investment. However, none of these three countries suffers severe food insecurity. Specifically, Russia is the largest recipient of agricultural investment and Russian per capita protein intake is 96 g/cap/day. China is the second largest destination country and China’s protein intake is 91 g/cap/day. As for the third largest destination country, USA, the per capita protein intake indicator is 112 g/cap/day. All of them are food secure countries and have per capita protein intake higher than 70 g/cap/day. When we zoom in on the food-insecure countries, the top three destination countries are India, Indonesia, and the Philippines, with investments of $9.2 billion, $8.0 billion, and $7.2 billion, respectively. Their average protein intake levels are only 58 g/cap/day, which is far less than that in China, the US, and Russia. The significant differences provide evidence that agricultural investment is distributed unevenly, with food-secure countries receiving a larger share of investment compared to food-insecure countries. Additionally, the size of the investment projects in food-insecure countries is relatively small.

## 5. Discussion on Investment Motivation

Since larger agricultural investment projects are barely found in food-insecure countries, we are interested in the investment motivation from enterprise perspectives. In this section, we discuss the investment motivation and hope to help food-insecure countries to attract agricultural investment to improve food security.

The International Production Compromise Theory [39] suggests three requirements for enterprises to conduct overseas investment: ownership, location, and internalization. Ownership refers to the special advantages that international enterprises usually own compared with other domestic enterprises in the host country, which would make up for their shortcomings when entering an entirely new market in the host country. The location suggests that international enterprises consider location factors when they invest abroad, such as production costs, transportation costs, market potential, and trade policy. Internalization represents the final requirement for international enterprises to conduct overseas direct investment. This means that international enterprises benefit from overseas investment more than directly transferring knowledge and technology. Among these three requirements, location advantage is the most crucial factor. It also explains the four types of investment motivation from the enterprise’s perspective: seeking new markets, seeking resources, seeking to improve production efficiency, and seeking special strategic assets.

### 5.1. Agricultural Investment Motivated by Seeking New Markets

The most common motivation behind conducting overseas investment is to seek new potential markets. The Factor Proportion Theory suggests that product prices affect factor prices and commodity trade can be a substitute for factor trade. In practice, differential trade policy in different countries could affect domestic factor prices, and therefore provide international enterprises with the opportunity to reallocate their supply chains across different countries [40]. New markets or potential customers are highly attractive for international enterprises when they conduct overseas investment projects [41]. Those countries with a high GDP growth rate are more likely to become the targets of overseas investors. In return, the investment would boost economic development in host countries.

For the top seven destination countries of aggregate agricultural investment, all of them have more than one trillion dollars in GDP. Moreover, four of them reached the level of $10,000 GDP per capita (shown in Table 4). In general, the size of the economy represents the current size of the market. International enterprises are more interested in a country with a large economy. The population is also important for enterprises to choose the destination, especially in countries undergoing rapid economic development. Likewise, the top seven destination countries of agricultural investment account for a large proportion of the global population. China has the largest population in the world (1.4 billion) and India’s total population reached 1.3 billion in 2019. The population in USA, Brazil, and Indonesia also exceeds 200 million. The huge population and economic development potential are commensurate with market potential. When an enterprise enters a new market at an early stage, it would expand easily with the growing market size and gain substantial long-term benefits.

However, the countries suffering from food insecurity are usually less developed countries, meaning that they have limited appeal for foreign investment [42]. In this case, non-government organizations (NGOs) should provide direct subsidies related to food and agriculture. The World Bank and other regional development banks are expected to launch some development projects aimed at improving the poor infrastructure in food-insecure countries. Additionally, we also hope that international enterprises could take more social responsibility to help the hungry people.

### 5.2. Agricultural Investment Motivated by Seeking Resource

Arable land is the key factor for agricultural production and is the motivation for many overseas investment projects [43]. With the global population growing and arable land resources becoming increasingly imbalanced, the food supply chain is facing immense pressure [44]. As the largest source country of agricultural investment, USA mainly invests in China, Canada, Mexico, Russia, and Brazil. These countries have a high growth rate of economic development, stable political systems, and abundant natural resources. The ratio of American investment in the production and processing sector is 3:2. By contrast, China’s overseas agricultural investment mainly concentrates on crop production and dairy products due to the scarcity of arable land resources in China. Approximately 50% of China’s overseas agricultural investment is directed towards Africa [38,45]. But there are concerns about the investment risks and debt sustainability of China’s large-scale overseas investment, as argued by Bandiera and Tsiropoulos [46].

It is true that investors may face more uncertainties when they choose food-insecure countries as investment destinations. The level of institutional governance, protection of property rights, and the existence of corruption would impact the investment decisions of foreign investors [47,48,49]. To improve the investment environment, governments in these food-insecure countries should establish effective and transparent regulatory systems to attract investors [49]. At the same time, they could implement the laws and suitable monitoring systems to protect foreign enterprises. An inclusive, friendly, and efficient investment environment is crucial for overseas agricultural investment to thrive.

### 5.3. Agricultural Investment Motivated by Seeking Efficiency

Efficiency-seeking guidance refers to reducing costs and pursuing higher profits by conducting overseas investments. It can be explained by the Product Life Cycle Theory, which suggests three development stages: the innovation stage, the maturity stage, and the standardization stage. At the innovation stage, enterprises focus on research and development (R&D) to introduce new production technology, improve machinery, and cultivate new varieties. To promote R&D and reduce innovation costs, the international enterprises usually locate their R&D departments in developed countries to take advantage of their dense knowledge and technology [50]. Moreover, financial markets in developed countries can also provide adequate financial support for international enterprises. During the maturity stage and standardization stage, production factors, such as natural resources and labor, become more important than knowledge and technology, especially in the agricultural sector. Therefore, emerging economies with large and cheap labors, such as China and India, become popular destinations of agricultural investment. Besides, both China and India have a relatively inclusive investment and business environment and therefore stay at the top of the destination list.

However, most of the food-insecure countries are African countries and struggle to provide qualified labor or other elements that could help enterprises to reduce costs. To increase attractiveness, these countries could improve infrastructure, offer special policy support, and assist international enterprises in farmer training. Besides, local governments need to control potential investment risks and maintain a safe and friendly investment environment.

### 5.4. Agricultural Investment Motivated by Seeking Strategic Assets

Strategic assets play a crucial role in the long-term development for international enterprises. The strategic assets include the sales networks, management experience, brand reputation, and globalization. Seeking strategic assets could explain some agricultural investment that flows from developing countries to developed countries. For example, some enterprises locate their innovation departments in developed countries to improve research efficiency. It is also consistent with the efficiency guidance. Some overseas investment projects are responding to the national development strategy [38]. For example, an increasing number of Chinese enterprises establish overseas production bases in Africa and Western Asia after the Belt and Road Initiative, which helps them to obtain some support in the domestic market [51]. Countries facing severe food insecurity should cooperate with other countries or international organizations to attract foreign investment and enhance their ability to provide strategic assets.

## 6. Conclusions and Policy Implications

The sources of global agricultural investment mainly concentrated in developed countries. The largest source country is USA, accounting for almost 20% of global agricultural investment. The majority of agricultural investment is contributed to by several countries. There has been an increasing diversity of source and destination countries in the past decade, which to some extent indicates a more inclusive investment environment worldwide. However, the global distribution of agricultural investment is uneven. Food-insecure countries, which are defined as countries with a per capita protein intake below 70 g/cap/day, only receive 20% of the global agricultural investment. Most investment in food-insecure countries are small-scale while larger agricultural investment projects are typically located in food-secure countries. The top three destination countries of agricultural investment, Russia, China, and USA, have a relatively high level of food security (around 100 g/cap/day on average). By contrast, countries suffering from severe food insecurity receive less investment. Among food-insecure countries, the top three destination countries are India, Indonesia, and the Philippines, with protein intake levels are only 58 g/cap/day on average, far less than that in Russia, China, and USA.

From the perspective of investment motivation, attracting overseas agricultural investment driven by many socio-economic factors, including the size of the population, the rate of economic growth, market potential, natural resources related to agriculture, the amount of labor, the level of knowledge and technology, and the possibility of obtaining strategic assets. Driven by different types of investment motivation, international enterprises determine whether to invest in developed or developing countries. Unfortunately, it is hard for countries suffering from severe food insecurity to provide attractive offers for overseas investors.

To address this issue, various stakeholders from all levels of society should work together to help improve the food and nutrition status in these countries. NGOs could offer more direct subsidies related to food and agriculture for food-insecure countries. We hope that international enterprises could take more social responsibility to help the hungry people. Especially during the recovery of the global economy after the COVID-19 pandemic, an increasing number of people are facing poverty and food insecurity. It is hard for them to guarantee their own access to adequate food and nutrition. The governments in these food-insecure countries should establish effective and transparent regulatory systems to reduce potential investment risks. Additionally, it is also important to improve infrastructure, provide special policy support, and assist international enterprises in farmer training, which would create a safe and friendly investment environment. The World Bank and other regional development banks are expected to launch more development projects to improve the poor infrastructure in food-insecure countries. Combining the efforts worldwide, we hope to achieve the SDG Target 2, ending hunger by 2030.

## Figures and Tables

**Table 1:** Top 10 source and destination countries of aggregate agricultural investment.

Source Country	Capital Investment (USD, Billion)	Percentage (%)	Destination Country	Capital Investment (USD, Billion)	Percentage (%)
USA	52.87	19.08	Russia	26.83	9.68
Switzerland	23.91	8.63	China	23.22	8.38
Germany	23.41	8.45	USA	22.07	7.96
UK	17.78	6.42	UK	12.15	4.39
Japan	14.37	5.18	India	9.22	3.33
China	13.37	4.83	Brazil	8.95	3.23
France	11.81	4.26	Indonesia	7.95	2.87
Netherlands	7.65	2.76	Mexico	7.94	2.87
Thailand	7.63	2.75	Poland	7.38	2.66

Data is collected from the fDi Markets database.

**Table 2:** Top 10 source and destination countries in the agricultural processing field.

Source Country	Capital Investment (USD, Billion)	Percentage (%)	Destination Country	Capital Investment (USD, Billion)	Percentage (%)
USA	19.65	19.24	USA	8.98	8.79
Germany	18.04	17.65	Russia	8.15	7.98
Switzerland	7.13	6.98	China	7.57	7.41
UK	6.55	6.41	U.K.	6.57	6.43
Japan	5.53	5.41	India	4.79	4.69
Italy	3.77	3.69	Germany	3.74	3.66
UAE	3.17	3.11	Poland	3.66	3.58
France	3.09	3.02	France	3.49	3.42
Mexico	2.87	2.81	Mexico	3.46	3.39

Data are collected from the fDi Markets database.

**Table 3:** Top 10 source and destination countries in the agricultural production field.

Source Country	Capital Investment (USD, Billion)	Percentage (%)	Destination Country	Capital Investment (USD, Billion)	Percentage (%)
USA	33.22	18.99	Russia	18.68	10.68
Switzerland	16.78	9.59	China	15.65	8.94
China	11.38	6.51	USA	13.09	7.48
UK	11.23	6.42	Philippines	6.58	3.76
Japan	8.84	5.05	Indonesia	6.09	3.48
France	8.73	4.99	Brazil	5.67	3.24
Thailand	6.77	3.87	UK	5.58	3.19
Vietnam	6.42	3.67	Romania	5.19	2.97
Malaysia	5.70	3.26	Vietnam	5.11	2.92

Data is collected from the fDi Markets database.

**Table 4:** The GDP and arable land area in the top seven destination countries.

Country	Received Investment 2003–2019 (USD, Billion)	GDP in 2019 (USD, Trillion)	Population in 2019 (Million)	GDP per Capita in 2019 (USD)	Arable Land in 2019 (1000 ha)	Arable Land per Capita in 2019 (ha)
Russia	26.8	1.69	146	11,617	121,649	0.834
China	23.2	14.70	1453	10,110	119,474	0.082
USA	22.1	21.37	334	63,953	157,736	0.472
UK	12.2	2.86	66	42,784	6086	0.091
India	9.2	2.85	1383	2061	155,369	0.112
Brazil	8.9	1.88	211	8884	55,762	0.263
Indonesia	8.0	1.12	269	4151	26,300	0.098

Data are collected from the fDi Markets database and FAOSTAT.

## Data Availability

The data can be found from fDi Markets database https://www.fdimarkets.com/ (accessed on 14 September 2021).
